# An Analysis of the Interaction Between Intelligent Software Agents and Human Users

**DOI:** 10.1007/s11023-018-9479-0

**Published:** 2018-09-25

**Authors:** Christopher Burr, Nello Cristianini, James Ladyman

**Affiliations:** 1 Department of Computer Science, University of Bristol, Merchant Venturers Building, Woodland Road, Bristol, BS8 1UB England, UK; 2 Department of Philosophy, Cotham House, University of Bristol, Bristol, BS6 6JL England, UK

**Keywords:** Artificial intelligence, Machine learning, Human–computer interaction, Nudge, Persuasion, Autonomy

## Abstract

Interactions between an intelligent software agent (ISA) and a human user are ubiquitous in everyday situations such as access to information, entertainment, and purchases. In such interactions, the ISA mediates the user’s access to the content, or controls some other aspect of the user experience, and is not designed to be neutral about outcomes of user choices. Like human users, ISAs are driven by goals, make autonomous decisions, and can learn from experience. Using ideas from bounded rationality (and deploying concepts from artificial intelligence, behavioural economics, control theory, and game theory), we frame these interactions as instances of an ISA whose reward depends on actions performed by the user. Such agents benefit by steering the user’s behaviour towards outcomes that maximise the ISA’s utility, which may or may not be aligned with that of the user. Video games, news recommendation aggregation engines, and fitness trackers can all be instances of this general case. Our analysis facilitates distinguishing various subcases of interaction (i.e. deception, coercion, trading, and nudging), as well as second-order effects that might include the possibility for adaptive interfaces to induce behavioural addiction, and/or change in user belief. We present these types of interaction within a conceptual framework, and review current examples of persuasive technologies and the issues that arise from their use. We argue that the nature of the feedback commonly used by learning agents to update their models and subsequent decisions could steer the behaviour of human users away from what benefits them, and in a direction that can undermine autonomy and cause further disparity between actions and goals as exemplified by addictive and compulsive behaviour. We discuss some of the ethical, social and legal implications of this technology and argue that it can sometimes exploit and reinforce weaknesses in human beings.

## Introduction

The software running various aspects of the world wide web plays an important role in shaping human behaviour in the growing list of essential and recreational activities migrated online. Engaging in these activities often requires interaction with software agents, which mediate between people and the content they seek (e.g. news, entertainment, shopping). There exist many types of software agents that people now interact with on a daily basis (e.g. recommendation engines for news or entertainment, wearable fitness devices, video games), and that are designed to pursue their own goals *autonomously*, in the sense that they are the locus of a decision-making process that is designed to pursue some goal.

In this paper we consider various scenarios that arise when a type of software agent, which we call an *Intelligent Software Agent* (ISA), interacts with a human user. In doing so, we adapt Russell and Norvig’s (2010) definition of *learning agents*, and define an ISA as any program that can be described as having a model of its environment, which it uses to take actions that enable the ISA to achieve its goals, while also acquiring further information that it can use to update the parameters of its model (see also Cristianini 2010). In the cases we consider, the environment of an ISA includes the behaviour of a human user, and the ISA’s goals depend on whether the interacting user performs certain actions (e.g. clicks, purchases, actions in a game, or physical exercise). Hence, what rewards the ISA obtains is conditional on its ability to influence the behaviour of a human user.^1^

Using language borrowed from control and game theories, we present a simplified model of autonomous behaviour, based on the classic work on “bounded rationality” (Simon 1956), in order to organise a variety of everyday interactions between ISAs and human users into a cohesive framework, which elucidates the differences and similarities of the subtypes of interactions. There are three features of all the subtypes we consider:

1. The ISA has to choose from a set of available actions that bring about interaction with the user. For example, recommending a video or news item; suggesting an exercise in a tutoring task; displaying a set of products and prices, and perhaps also the context, layout, order and timing with which to present them.
2. The user chooses an action, thereby revealing information about their knowledge and preferences to the controlling agent, and determining the utility of the choice the ISA made.
3. The cycle repeats resulting in a process of feedback and learning.

Although we focus on a single special case (i.e. interaction between an ISA and human user), our discussion also applies more generally, especially to other cases of control and interaction.^2^

The type of interaction at question is behind the revenue model of many companies, and is highly complex since the environments of ISA’s are human minds, which depend on culture. Extensive research and development has generated increasingly effective systems that learn from experience. A typical ISA, such as a recommender system, might have to select a set of videos for a user to watch (out of a vast catalogue), using any available information or signal it has about the given user (e.g. location, time, past usage, explicit ratings, and much more). In this case, the ISA’s goal is to select an action that, for the given user, maximises the expected *click*-*through rate* (CTR): an expression of the probability of users clicking through links. The user, on the other hand, has the option of clicking on one of those videos, or opting out, and their utility function might be related to some entertainment or information need.

There are much more complex utility functions and decision procedures than this. For example, the many ISAs behind Google’s advertising network select advertisements, out of catalogues of millions, to show on web pages (O’Reilly 2015). A particular advertisement is selected by an ISA autonomously running an auction that determines which advertiser is willing to pay the most for their advertisement to be shown on this occasion. This list of possible bids is simultaneously weighted against a single “quality score”, which is obtained by estimating the probability that a given user (described by thousands of possible signals) would click on each of the possible advertisements, and combining it with the potential price of each possible click—this can be idealised as the ISA computing an expected utility. The “quality score” is used to rank the various options (advertisements) and determine the optimal decision—in this instance, a combination of the revenue associated with each advertisement, and the respective probability of click-through. All of this must happen within the *milliseconds* it takes for a web page to load (Google 2017). Another example is the score assigned to drivers and passengers in a ride-sharing app, acting as a proxy for the quality of service each party can expect from the other (e.g. cleanliness of car, friendliness of individual), and used to automatically match passenger with driver.

Machine learning has been used for years in these contexts, in which people interact with ISAs often without realising it, with significant success. However, there is confusion about how to think about these interactions. Our primary aim is to use a review of what is currently possible with persuasive technologies to develop a clear framework for thinking about the many ethical, legal, political, psychological and social issues that arise as a result of continued human interaction with ISAs. We do not presuppose or argue for claims about the right context, degree and extent of ISA use, but our framework facilitates discussion of the challenges that have emerged, and will arise from future technological innovation. According to Floridi and Sanders’ (2004) definition of a moral agent, ISAs are moral agents (Sect. 5.3). Our discussion is neutral about this, though not about whether ISA’s are agents in the sense of making decisions in accordance with preferences based on a representation of the environment (as in the definition above and detailed in the next section). As such their actions and their effects on human beings are certainly morally significant.

The structure of the paper is as follows. Section 2 introduces the concept of an ‘autonomous agent’, some relevant terminology, and a simplified model inspired by key concepts from bounded rationality. Section 3 adds the requirement that the ISA’s reward depends on the actions taken by the human user (as in 2 above), deducing from the resulting framework a simple taxonomy of the different ways in which the ISA can influence its human user. Section 4 uses this framework to review various situations that can be described in terms of persuasive interactions and introduces a number of questions about second-order effects, such as belief change and behavioural addiction, which may arise from unintended feedback loops. Section 5 outlines a series of ethical implications, discussing how recent proposals fit within our framework, and concludes.

## Autonomous Agents and Bounded Rationality

We begin by describing the concept of an *autonomous agent*, tasked with choosing some action that depends on the state of its environment (which could include another agent), before showing how this general description applies to the ISA-human user interactions. The description is an idealisation, and as such does not consider details of the physical implementation of the agents in question (e.g. whether they are biological, social, software, hardware, or even a mixture).

The need to describe or model the choice behaviour of an autonomous agent arises in many domains. The simplest models assume an agent trying to pick the best action in a given state, where “best” refers to whatever maximises *expected utility* or *reward.*^3^ The agent can (partially) observe the current state of the environment, compute the probability for each of the possible outcomes of each action, and then evaluate each outcome on the basis of its expected utility. However, real agents do not necessarily know all the logical consequences of all their actions; they must compute them (and their probability) within various constraints (e.g. computational tractability). As such, the standard concept of an autonomous agent incorporates the notion of `bounded rationality’ (Simon 1956), but still remains highly idealised (Gigerenzer and Selten 2002). We also adopt the perspective of bounded rationality, as it allows us to establish some terminology and distinguish between different types of cases that are otherwise easily confused. For the purposes of our analysis and discussion (Sects. 4 and 5), and to help draw attention to specific features of our conceptual framework, we present a simplified (and idealised) model of a bounded agent interacting with its environment, where the ‘environment’ is restricted to the perceived actions of another interacting agent (see Russell and Norvig 2010, for further discussion on these concepts). This level of abstraction allows us to focus on specific behaviours of the interacting agents and their environment, while screening off irrelevant details. For example, we do not consider questions such as whether the probabilistic model of the environment is the same for both agents, and we ignore details such as whether the interaction is mediated by a device such as a smartphone or computer screen. Although these details may have important consequences worthy of exploration, in this paper we aim for a more general level of analysis.

Consider an agent that must choose an action from a set of possible actions (a∈ A),^4^ while its environment is in a particular state (s_i _∈ S). The outcome of this action is a change in the state of its environment (s_j _∈ S), which may not depend entirely on the agent’s actions. Agents assign numerical values to outcomes (U: AxS → ℝ), to indicate the expected utility associated with them, and the goal of the agent is to choose the action that will maximise its overall *subjective utility* (hereafter, utility). The agent knows the utility for each outcome, given by U(a, s_j_), and has a probabilistic model of the possible consequences of its action, given by P(s_j_ | a, s_i_): the conditional probability that by performing action a in state s_i_, the state of its environment will change to s_j_. Typically, this is solved by calculating the expected utility of taking some action a in the current state (s_i _∈ S), then averaging over all possible outcomes (s_j _∈ S):

$$EU\left( {a, s_{i} } \right) = \mathop \sum \limits_{j = 1}^{n} P(s_{j} |a, s_{i} ) U\left( {a, s_{j} } \right)$$

and repeating this for each possible action (a∈ A), before selecting the action with the maximum expected utility:

$$a^{*} = \arg \max_{a \in A} EU\left( {A, s_{i} } \right)$$

This simple model separates the beliefs of the agent (i.e. its probabilistic model of its environment) from its goals (i.e. the utility function), as well as allowing us to combine them into a single function that can be used to represent its preferences.

Importantly, the model involves steps where computational and other resources are needed (e.g. calculation of expected utility), and this serves to differentiate ways in which agents deviate from “rational” behaviour. The most common cases involve limitations concerning information about S, the computational cost of maximisation, constraints upon memory, and knowing (or accessing) all possible actions in A (leading to various models of bounded rationality, e.g. Simon 1956; Rubinstein 1998; Gigerenzer and Selten 2002, which adapt the above definition in different ways). These limitations apply to both artificial agents and human users, and both use heuristics—of very different kinds—to bypass enumeration of all possible outcomes and exhaustive deliberation. Heuristics are often approximations that only work in a specific, and narrowly defined environment, limiting their use. As discussed in Sect. 4.3, by knowing the biases and heuristics used by an agent, it is possible to steer their behaviour away from a more rational outcome.

The special case we consider is when part of the environment is another agent who is guided by a distinct utility function.^5^ The controlling agent attempts to steer the behaviour of controlled agent, typically towards states that maximise the expected utility of the controlling agent. If the utility function of the controlling agent is aligned with that of the controlled agent, the interaction is one of cooperation; to the degree that they are not, there is an element of competition (e.g. in games such as Chess or Go, and a wide variety of other cases where two agents pursue incompatible goals while interacting). This control problem is in general probabilistic because there is no guarantee that the attempted control will be successful in bringing about the desired outcome. The controlling agent bets on the outcome of its actions, because it can only probabilistically predict the reward they will generate.

We consider the case where the controlling agent is an ISA and the controlled agent is a human user. Some ways for an artificial agent to control the actions of a human user include: to offer incentives to perform a given action (e.g. a recommender system presenting a highly rewarding news item or video to a user); to exploit some heuristic shortcuts of the user (e.g. a recommender system using social pressure to encourage the user to share a news item); to deceive (e.g. a recommender system resorting to clickbait to increase its utility without the user benefiting); and/or to coerce (e.g. a recommender system requiring an advertisement to be watched as a prerequisite for use). We can describe all of these sub-cases, using the simplified model presented in this section, applied to both an ISA and a human user, and also separate them according to how they operate by considering different components of the model (Sect. 3).

Various disciplines have focused on different aspects of the general problem above. For example, in classical control theory, the controller can observe the state of the controlled system, and knows the actions it can take, in addition to their effect on the state of the controlled system. Here, the control problem is to select the most rewarding action for the controlling agent, where the reward is defined in terms of the state of the controlled system (see Sutton and Barto 1998). In economics and decision theory, a rational agent can observe its own state and that of its environment, and has a probabilistic model of the effect of its actions on the state of the environment (see Binmore 2009). Knowing also the utility of each state of the environment, the rational agent’s goal is to select the action with the highest expected utility. Behavioural economics assumes that an agent’s rationality is bounded, and emphasises an agent’s use of heuristics: shortcuts that can approximate good decisions in typical settings, but may lead to irrational behaviour in non-typical domains (Kahneman 2011). Finally, in Artificial Intelligence (AI) the artificial agent needs to learn the effect of its own action, and their utility, while at the same time trying to control its own environment (see Russell and Norvig 2010). Modern AI agents often use an architecture that does not enumerate all outcomes of all actions, but rely on model-free algorithms (e.g. Q-learning) to estimate the expected reward of an action, without exploring all possibilities—this is equivalent to using heuristics. Similarly, when modeling an artificial agent, it is often sufficient to estimate their utility function, without distinguishing between goals and beliefs.^6^

Most AI agents learn their heuristics from interactions with their environment, and these heuristic functions can be iteratively tuned by using machine learning algorithms (e.g. reinforcement learning techniques such as bandit algorithms), in combination with large amounts of training examples (Sutton and Barto 1998).^7^ This is the case for many of today’s AI (and learning agents more generally), which constantly refine their heuristics, learning to predict the expected reward of their actions by constantly looking at their outcomes.

There are many different processes that can be used in practice to design an artificial agent. While its utility function is selected by the designer and kept fixed, its beliefs (model of the environment) can either be directly designed, fine-tuned by experimentation [e.g. A/B testing: a standard technique similar to running a clinical trial on a sample of users, which was famously used by Google to determine which of a possible 41-shades of blue to use for their web links (Arthur 2012)], or directly learned by machine learning methods [e.g. by reinforcement learning procedures, which allow an actively deployed artificial agent to improve constantly, while tracking drifting environments (Sutton and Barto 1998)]. In all these cases, it is possible that the design process stumbles upon a highly effective heuristic, and then exploits it, or improves on it through further exploration. Except perhaps for the case of explicit design, there is no reason to expect an artificial agent to actually compute the outcomes of each possible action and their reward—effective behaviour can often result from simple rules of thumb or heuristics. This might also make it difficult to interpret by humans (Burrell 2016), and has additional implications for our discussion in Sect. 5. As mentioned above, on the Floridi and Sanders (2004) definition, the class of moral agents extends to the kinds of artificial agents considered in this paper. Section 5.3, briefly presents their proposal which allows that moral agents may nevertheless be “mind-less”, in the sense of not necessarily exhibiting mental states (e.g. emotions) or responsibility (i.e. ‘mind-less morality’). This paper shows that whether or not ISAs are properly considered to be moral agents, their integration with human action raises moral challenges.

## Intelligent Software Agents and Human Users

In this section, we consider the special case of interaction between ISAs and human users in more detail. An ISA is usually deployed for the purpose of maximising a given utility function that reflects the goals of whoever developed it, for example, to increase the revenue of a company or spread a political message. The ISA chooses from a set of actions whose outcomes partially depend on the choices made by the human user. This means that depending on the relation between the two utility functions, the ISA might effectively be *collaborating* or *competing* with its human user—an observation related to the *value alignment problem*^8^ (Sect. 5.1). There are various ways in which the ISA can increase its utility by inducing the user to perform certain actions (e.g. reading or sharing an article). In particular, the actions of the ISA (e.g. selecting an article) are divided into three subtypes: *coercion*, *deception* and *persuasion*. This section introduces our framework, and briefly introduces each of the three subtypes. Section 4 explores some of them in much more detail.

To simplify the discussion, we focus on the running example of *recommender systems,* such as those commonly used for news and videos, but also in the context of video games, fitness devices, and various other interfaces. A recommender system (RS) is a type of interface that mediates the interactions of an ISA and a human user and is largely (if not entirely) under the control of the ISA. In these interactions, we assume that the user has a particular need (e.g. a specific type of information or entertainment), and the ISA responsible for selecting the recommendations has the goal of increasing some form of engagement (e.g. clicks, shares, likes, etc.). The user’s engagement is interpreted as signalling the relevance of the recommendations, and is therefore considered a rewarding (and informative) state by the ISA. We use ‘click through rate’ (CTR) as a running example of a measure of reward used by the ISA, but any other measure of ‘success’ could be used. In this example, the ISA can recommend a selection of relevant items from a large catalogue, along with supporting meta-information (e.g. images, descriptions, ranks, etc.), which are designed to help the user make a decision. The user also retains the choice to leave at any given time, which would not normally be in the interest of the ISA.^9^ The popularity of RSs is largely due to their ability both to increase the utility of boundedly rational users—who would otherwise be unable to examine each item in the catalogue on their own—and also maximise the ISA's utility. Importantly, one can describe the goal of the ISA as either ‘maximising the relevance for the user’, or as ‘maximising the CTR’. These two quantities are often conflated in the technical literature, but they are not necessarily aligned. As discussed in Sect. 5, there is an important distinction between short-term and long-term benefits for the human user, and a hidden cost of using proxies for utility.

### Coercion and Deception

We briefly consider below *coercion* and *deception* which are viable and popular methods to control the behaviour of a user. Coercion involves denying access to or imposing conditions on certain actions (a∈ A), whereas deception affects the way in which the user assesses the utility associated with some state of the world, or the payoff of the choice (i.e. they still act rationally, but on false premises).

#### Coercion

A familiar, mild form of coercion is when the user is in need of information and so accesses a RS, but cannot skip a promotional video. Since they have no choice (i.e. their set of actions have been restricted), and were not looking for that video, they have been *coerced* to see it. This situation is also commonly found in a variant form: when other actions are requested as a prerequisite to gaining access to some service (e.g. sharing personal data in order to gain access).

In some cases, it may be not be straightforward to determine whether some action constitutes coercion. Consider a recent case in Germany, in which an antitrust lawsuit targeting Facebook raised questions as to whether the social media company used its popularity to coerce users into agreeing to their terms and conditions governing personal data collection. As part of this investigation, EU antitrust regulators considered whether the practice of using the fear of social isolation constitutes a form of extortion, or whether it is merely part of the terms of use for the social media platform, which users are free to reject (White and Matussek 2017).

#### Deception

As a particular type of deception, consider the case of clickbait, or an ISA that selects links with misleading descriptions. Clickbait and phishing scams are a form of deceiving recommendations, which misrepresent their real contents, sometimes requiring several additional links before that is discovered. The user will click on them, thereby increasing the utility of the ISA (e.g. increased CTR), but have themselves been mislead, and their own utility has not been increased (e.g. they do not in fact obtain relevant information). Other forms of deception might include unwanted pop-ups warning of a virus threat; advertisements that offer a software package for one purpose, only to deliver something else (e.g. malware); or harmful attachments to emails.

### Persuasion

In cases of persuasion, the general setting is probabilistic. The ISA controls which options are presented, based on its probabilistic beliefs about the actions taken by the user (or group of users) in response, as well as its own goals. In line with the usage in persuasive technology (Fogg 2003), we define `persuasion’ as any case where the ISA is influencing the user’s actions without using *deception* or *coercion*. We introduce and distinguish two important cases of persuasion: *trading* and *nudging*.

#### Trading

Trading occurs when the ISA has some knowledge of the user’s goals (either revealed by their actions, or explicitly stated by the user), and presents the user with options that are expected to increase both the user’s utility and the ISA’s utility—both sides benefit from the trade. The ISA models the user’s utility, but it does not adopt that model as its own. The ISA’s goal is to maximise its own utility, and the increase in the user’s utility is a means to that end. For example, the goal of a news site may be to increase its traffic, and so it will offer articles that are most valuable to the user and most likely to be clicked on. But it is also possible to consider cases where the goal of the ISA is to make a profit, while also finding a good deal for the user, and so this would imply more complex trade-offs (e.g. Google’s advertising system). Trading can also involve altering the payoff structure of the choice through financial incentives (e.g. offering a discount to persuade the user to purchase an item), as is done in dynamic pricing (Ezrachi and Stucke 2016). Often, the main benefit for the user is to be able to efficiently search large sets of options at a low cost, in this way making a more rational choice.

In Sect. 4.2 we describe in more detail some specific cases of real-world trading by ISAs. We discuss how ISAs segment users into various groups, as a prerequisite for modelling a user’s utility, and how this enables trading to be carried out between user and ISA.

#### Nudging

Nudging is when the ISA seeks to influence the human user’s decision by targeting the user’s biases and heuristics. Unlike trading, nudging does not change the financial incentives for the user, and may or may not increase the user’s utility (Thaler and Sunstein 2008). When the user’s biases are predictable, as many are (Ariely 2008), they can be exploited by nudges that steer a user towards actions that they may not rationally choose otherwise and might even be of detriment of the user. For example, in the familiar case of recommendation, an ISA might try to induce users to click on an item that is sub-optimal for the user, but preferred by the ISA, by using emotional associations, social effects, or other methods to bias their choices. This would not constitute *deception*, because the ISA is not *misrepresenting* the options.

It is important to note that a user can act rationally while still decreasing its own utility (e.g. if they operate under false beliefs or incomplete information). In this case, a nudging agent might steer the user towards better options, as is the case for some type of fitness devices or health interventions. Section 4.3 discusses how nudging can play a positive role when users have conflicting sets of internal goals, and may even be used to promote a user’s autonomy. When used against the interest of the user, however, nudging can reduce their autonomy and utility and this needs to be considered by regulators.

### Second-Order Effects

Second-order effects might arise unintentionally when the user’s beliefs or utility function are modified as a result of long-term interaction with an ISA. Rather than focusing on the ways in which the ISA can affect individual actions in the short-term, as described in the previous sections, we consider more permanent changes in the user resulting from the prolonged interaction with the ISA. These second-order effects can arise because human users are also learning systems, constantly revising their models, beliefs and expectations through trial and error (e.g. reinforcement learning). The learning dynamics of the cases we consider, may include positive feedback loops, which could amplify some of the smaller effects over longer periods of time (Sutton and Barto 1998).

Section 4.4 considers how repeated exposure to certain types of content can result in changes to a user’s belief system (e.g. their assessment of what the mainstream consensus is on a given topic; Sect. 4.4.1). Repeated exposure to a rewarding stimulus may generate behavioural addiction in human users, and result in a change to their utility function (Sect. 4.4.2). Therefore, while pursuing some short-term goal, an ISA might end up changing not only the user’s immediate actions (e.g. whether to share a news article or watch a video) but also long-term attitudes and beliefs, simply by controlling the exposure of a user to certain types of content.

The types of interaction between an ISA and a human user introduced in this section can be accommodated within the taxonomy outlined in Box 1. Section 4 reviews various techniques used in persuasive technologies, informed by this taxonomy.

**Box 1:**
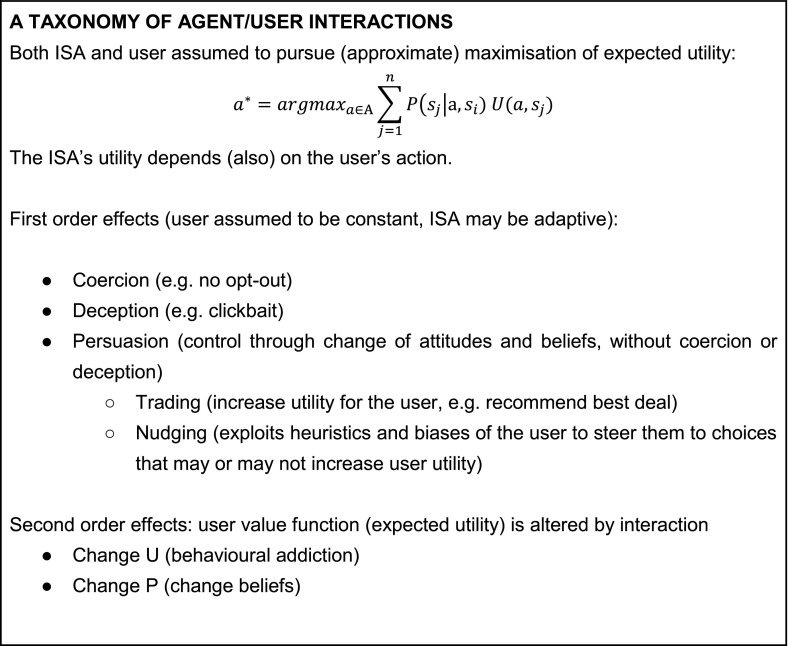
A summary of the taxonomy developed in the paper, and the types of interactions that can take place between an ISA and a human user. The interactions are structured by how the components of the Maximum Expected Utility formula are involved in the control process

## Persuasion of Human Users by Intelligent Software Agents

This review of *persuasion* is informed by work being done under different labels within different communities, such as persuasive technology (Fogg 2003; de Vries et al. 2017); persuasion profiling (Kaptein et al. 2015); gamification (Deterding et al. 2011); habit-formation (Eyal 2014); and influence (Cialdini 2009). This section reviews some of the methods currently used by ISAs to persuade users guided by the taxonomy outlined in Sect. 3.

### Feedback

It is important for an ISA to have information about the goals or needs of the user. While it is possible to ask them (e.g. to rate or review various products), it is often more economical to just observe their behaviour and collect information about what they end up clicking on. In online interactions, a user’s actions are monitored by the ISA as a process of ongoing *feedback*. In general, feedback is a signal going from the controlled system (e.g. human user) to the controller (e.g. ISA), which can enable the controller to determine and learn from the consequences of its actions (i.e. revising how the controlling agent classifies some behaviour). In the case of learning agents, the feedback plays another role: they can use it to adapt their models of the user, so as to behave more effectively. For example, in a recommender system (RS) for video content, the ISA proposes a few options to the user, then the user makes a choice, providing feedback to the controlling agent in the form of new information about the world, as well as the reward (if any) that has been received.^10^ The process of a controlling agent acting on the environment, and the environment feeding back some information about its new state, causing the controller to consider a new set of actions, is described by classical control theory, and is also known as a *feedback loop*.

Feedback loops are processes commonly found in biological and sociological systems, as well as other physical systems. *Positive feedback loops* are processes in which a change in one part of the system increases the magnitude of another part, which in turn intensifies the original cause (i.e. a change in A increases B, which in turn increases A). Positive feedback amplifies causal loops, which in extreme cases can push the system far from a stable equilibrium due to self-reinforcing dynamics (e.g. live audio feedback). For example, watching a recommended video may increase the need to watch another video (e.g. through advertisements, suggestions and preloading techniques), and users may end up watching more videos than intended due to unexpected levels of reward. In contrast, a *negative feedback loop* is a process in which a change in one part of a system acts on another part of the system so as to return the variable of the initial part to its original state, thereby maintaining the equilibrium of the system. For example, when a search engine correctly answers the query of a user, thereby satisfying the user’s information need and closing the interaction. This topic is discussed further in Sects. 4.4 and 5.

A recent article (Kramer et al. 2014) created some controversy when it reported that the emotional state of Facebook users could be estimated from their choice of words, and that this could be influenced by selectively exposing those users to different kinds of content. While this was done only in one iteration, and not for a sustained time, positive feedback loops might amplify the emotion of the most susceptible users.

Typically, what the ISA considers as feedback is not generated explicitly by the user (e.g. rating recommendations), and so ISAs often make use of implicit feedback instead by evaluating behavioural signals in the user’s interactions (e.g. interpreting a click as an indirect sign of approval, or relevance). These “digital traces” allow the ISA to update its model about what the user enjoys watching—in the context of information retrieval (as for news or videos searches) this is called “relevance feedback” (Baeza-Yates and Ribeiro-Neto 2010).

Users might be unaware that some of their actions can be interpreted as indications of the content’s relevance (e.g. likes, shares, clicks). A recent study (McDuff et al. 2013) demonstrated that a user’s facial responses to media could be used to automatically determine whether they “liked” the media, or had a “desire to view again”, potentially allowing ISAs to quantify the effectiveness of their actions (e.g. selecting advertisements or other media) by gathering implicit feedback. It is now possible to infer ‘private’ information about the user (Kosinski et al. 2013; Matz et al. 2017) from their online actions, since some of them (e.g. “likes”) correlate strongly with personal traits including: sexual orientation, ethnicity, and political views (e.g. Kosinski et al. 2013), and this raises a number of ethical concerns (see discussion in Sect. 5).

By using CTR as a proxy for relevance feedback, it becomes possible for the agent to learn what makes the user click, without disturbing the user with questions. However, as a result the ISA will not know why the corresponding item was considered rewarding to the user; all it will know is that it was considered rewarding enough to click. As such, it is not clear that the ISA is able to learn the “real” wants and needs of a user, as opposed to simply what makes them click. This could make the value alignment of an ISA and user’s goals increasingly difficult. The problem is exacerbated in cases of nudging, where it might become possible that the ISA ends up exploiting psychological vulnerabilities, rather than proposing trades that are actually beneficial for both parties. Related concerns have been raised by some technology designers (Lewis 2017), and will be further discussed in Sect. 5.

### Trading

The idea behind trading is that the ISA has some knowledge of the utility function of the user, possibly because the user has declared it explicitly (e.g. a hotel booking website might ask the user to specify if they are more interested in location or price), the ISA has inferred it by observation (e.g. a RS used by a social media platform will try to infer the interests of the user based on their online choice of news), or the designers have hardwired their assumptions about it into the ISA (e.g. a price comparison site might assume that the user will prefer the cheapest options). A mix of the above is also often used.

Consider the RSs used by social networks, news and entertainment websites, and online shops. With knowledge of the user’s utility, an ISA will be able to trade by offering a deal to the user: relevant and useful information in return for clicks, or other forms of compensation, such as purchases (e.g. a video recommendation site offering both free and paid-for videos). Here, deception and coercion are not used, and the user benefits from the help offered by the ISA in locating valuable content. In trading, both the user and the ISA may increase their utility, and so they are not necessarily in competition with one another. However, tensions may arise if the ISA needs to select between a free video and one that requires payment, while calculating the expected revenue that might result from different advertisements associated with different videos. In this case, the utility functions of user and ISA may not align, and this is typical in offline instances of trading as well. An ISA may be able to exploit the informational asymmetry inherent in non-transparent markets due to large-scale (and real-time) data collection, in order to alter the cost associated with the recommended items (see Ezrachi and Stucke 2016). In some special cases the price can also be adapted to a particular user in real-time (i.e. dynamic pricing).^11^

This familiar situation fits within the simplified model introduced in Sect. 2: the user has a goal (e.g. consuming entertainment or information); the ISA has a goal (e.g. maximising CTR); the user has a set of actions to choose from (e.g. engage with certain items, or leave site); the ISA has a set of actions to choose from (e.g. select items, order items, present items in a particular manner); their respective beliefs (e.g. the ISA's model of the user’s own utility) influence which actions they each take; and both can use feedback to update their models of the world (e.g. the user may notice newer (more relevant) recommendations, as a result of an earlier purchase).

In such instances, the key benefit of trading with an ISA is that the system can help users remove the worst options from the list, and focus their resources to a narrower set of promising options. This may greatly benefit a boundedly rational user by helping ensure they act in accordance with their goals. This is the intended role of RSs found in news aggregators, social networks, online music shops, video websites (see Jannach et al. 2010; Ricci et al. 2011 for introductions).

In cases of trading, RSs often help users to save computational resources: the ISA typically recommends a ranked list of items, from which the user is expected to choose in order to maximise their utility. On the other hand, the close observation of large numbers of users provides ISAs with the tools to predict users’ future behaviour. Although large companies with significant traffic might create individual models for their users,^12^ a typical strategy for RSs is to identify *types of users* that are similar in some regard, and then recommend them similar items. This requires the ISA to segment users according to the relevant similarities. For example, an ISA could use traditional *demographic segmentation* to carve up a population (e.g. age, gender, income, job, or location), such that the segments are sufficiently homogeneous to enable recommendations that can benefit both the user and the ISA (e.g. an advertisement that is targeted at a particular age group, in a certain geographic location, which is correlated with high income). Alternatively, an ISA could use *behavioural segmentation* to group users based on similar behaviours (e.g. targeting people who bought a certain product, visited a certain website, or liked a particular post). Finally, the ISA could use *psychometric segmentation*, which groups users according to psychological traits (e.g. personality traits, or emotional state).^13^

Each of the above methods of segmentation allow RSs to employ a technique known as “collaborative filtering” (Ricci et al. 2011). This utilises the information analysable from segments of users to enable the RS to predict the future patterns of similar users. The benefit of collaborative-filtering techniques is that the RS does not need to ‘know’ anything about the content of the items—it is sufficient that some users prefer some items to others. Various algorithms can be created to partition the set of users and the set of items, creating classes of users that like the same type of items, and classes of items that are liked by the same type of users (e.g. neighbourhood-based algorithms).

Another way to make recommendations is to segment the list of items (e.g. different genres of books, similar music), and then recommend an individual user (or segmentation of users) ‘more of the same’. This is known as “content-based filtering”, as it requires segmenting the list of items based on the similarity of their content. The RS then selects items based on a comparison between the content of the items and some knowledge of the user’s (or segmentation of users) relevant preferences. The content of each item is represented as a set of descriptors or terms (e.g. the set of words that appear in a text, genre of music). Until recently content-based methods were costly for most datasets except text. However, with the rise of Deep Learning methods (e.g. Kingma et al. 2014; LeCun et al. 2015) it is now possible to make content-based recommendations of songs, images, videos, and more.^14^

It is also worth noting that similar methods can be used to mediate between multiple users of a platform (e.g. ride-sharing or room-sharing services), rather than between user and content. In these cases, users are ranked on the basis of aggregate feedback from the community (e.g. star ratings), such that a driver or host may be motivated to improve the level of service they offer, in order to maximise the probability that they receive a positive rating. This can help regulate the system without the need for top-down intervention (O’Reilly 2013), and can be viewed as a form of trading between the two user’s, which is mediated by an ISA.

Each of the above techniques allows ISAs potentially to recommend the best options available to the user (or users), while simultaneously increasing their own utility (including increased learning about the user’s goals). So long as the ISA can be carefully designed to exactly maximise a user’s utility, its recommendations can benefit the user. However, any misalignment between a user’s utility and that of the ISA represents a possible source of tensions and conflicts of interest as discussed further in Sect. 5.

### Nudging

Nudging occurs when an ISA attempts to induce user engagement without significantly altering the payoff structure of the options, and without coercion and deception (Thaler and Sunstein 2008). Nudging is successful because humans are limited in the resources they can devote to decision-making (e.g. time, information, and computational resources), and like artificial agents,^15^ human agents use heuristics to quickly select a few salient options when making decisions (Simon 1956; Gigerenzer et al. 1999). However, heuristics are often domain-specific, and may lead to irrational choices if used in more general domains. In the behavioural sciences, they are often attributed to the first of two separate systems that we use for judgment and decision-making: one that relies on habits, associations, and emotions to make fast decisions on the basis of limited information, and another for slower more deliberative decisions (Kahneman 2011).

Knowledge of how these predictable heuristics work (see Ariely 2008 for examples), allows others to exploit them, by influencing what is known as a ‘choice architecture’—a concept made famous by Richard Thaler and Cass Sunstein in their (2008) book ‘Nudge’. Thaler and Sunstein (2008) explore the various ways in which the choice behaviour of individuals can be affected by intervening on (i.e. “nudging”) how the various options are presented (i.e. the choice architecture).^16^ For example, altering the order or layout of choices alters an individual’s preferences (e.g. *framing* and *primacy bias*); deploying certain design features can increase the probability of some options being noticed and selected (e.g. *availability*); and altering the concepts associated with the presentation of a choice, can prime individuals to prefer certain options (e.g. *priming* or *representativeness*) (see Kahneman 2011).

Because of the popularity of this concept, there now exists a large number of nudges, which have been subjected to empirical study. Sunstein (2016, pp. 26, 27) provides a list of some of the most popular, which includes: *defaults* (e.g. having some option pre-selected, such that no intervention by the user still results in a choice); *simplification* (e.g. ensuring forms, such as financial aid, are easy to understand); *reminders* (e.g. timely reminders that bills or appointments are due); *personalisation* (e.g. a message that targets some personal characteristic of the user); *framing and timing* (e.g., by sending reminders and messages at a time when people are likely to be paying attention); *uses of social norms* (e.g., disclosure of how one’s energy use compares to that of one’s neighbors); and *precommitment strategies* (e.g. questions where people agree, in advance, to some course of action). However, despite the popularity and interest in nudges in general, there has been comparatively little attention paid to *digital nudges* in particular (see Mirsch et al. 2017; Weinmann et al. 2016 for two recent exceptions, and Yeung 2017 for a more general discussion).

Some digital nudges are simply online implementations of their physical counterparts, and do no more than reflect the intentions of their designers (Weinmann et al. 2016). However, others are refined by an ISA through relevance feedback, and adapt to different models of individual users. One particular *adaptive nudge*, which has grown in commercial popularity, is the use of psychometrics to infer (from behavioural signals) the personality traits of users, and to use this knowledge to design tailored content (Matz and Netzer 2017). The popularity of this nudge is due, in part, to empirical evidence that suggests interventions (e.g. messages or advertisements) are particularly effective when adaptively tailored to people’s unique psychological characteristics and motivations (Cialdini 2009; Matz et al. 2017).

A series of studies conducted online with 3.5 million individuals (Matz et al. 2017), investigated whether tailoring the content of persuasive messages (e.g. advertisements) to individuals’ psychological characteristics significantly altered their behavior. The authors found that messages tailored to an individual’s level of *extraversion* or *openness*-*to*-*experience* (two common personality traits) resulted in “up to 40% more clicks [measured by CTR] and up to 50% more purchases [measured by conversion rate] than their mismatching or unpersonalized counterparts” (Matz et al. 2017, p. 1). This is clear evidence of how a technique that is readily deployable by ISAs can significantly control a human user’s behaviour.^17^

Adaptive nudging can also be used to help user’s achieve their own goals. Consider the increased use of wearable technology for health and wellbeing. Many of these devices are designed to be worn 24/7, are equipped with a variety of sensors (e.g. accelerometers, bioimpedance sensors, and temperature sensors), and have access to huge streams of behavioural data. Such devices can make use of adaptive nudges that require *timely delivery*—a well-studied influence strategy (Cialdini 2009).^18^ Alternatively, an ISA may use player profiling in video games (i.e. constructing a model of the player’s behaviour) to predict a player’s actions (Bakkes et al. 2012), and then adapt the gameplay by nudging a player towards a particular goal (e.g. improving skills within the game), and trying to improve the overall user experience (Yannakakis and Hallam 2007).

With the rise in machine learning algorithms, these techniques can also be of wider benefit to technology designers. For example, some companies can compile vast datasets, consisting of millions of user’s behaviours, and learn how to improve their systems by conducting mass experimentation (Maher 2016). However, app developers can also use this feedback to monitor a user’s attention and engagement within a game, in order to adjust elements of the game’s mechanics just before they lose interest and quit the app—likely as a means of increasing exposure to pay-to-play elements that are the basis of many app developers’ business models (Byrnes 2015). Nudges that encourage excessive consumption of online entertainment need to be carefully studied, particularly if aimed at children or users’ prone to addictive behaviours (see Sect. 4.4.2).

The above examples are only a small sample of the digital nudges that are being tested and deployed online. Some are benign, but others raise ethical concerns that need further investigation. On this point, Thaler and Sunstein (2008) note that some type of choice architecture is unavoidable—one must always frame a decision problem in some manner, and in so doing, nudge users. The same applies to ISAs, some of which are able to alter the architecture to suit their goals (Hauser et al. 2009). If a choice architecture is always present, then it is unavoidable that an ISA is nudging a user in some way to better obtain its goals.

When an ISA has the capability to control aspects of a choice architecture, and learn which ones lead to increased reward, this may lead to the emergence of a positive feedback loop, which promotes not the most relevant offers for the user, but rather the most compelling—this scenario is in need of investigation.^19^ In Sect. 5.2 we will discuss some of the ethical and philosophical issues in respect of nudging, and its possible impact on user autonomy. Some have proposed using nudges to bypass the reactive system that most nudges often target, and instead raise the probability that a user will decide in a more deliberative and rational manner (Levy 2017). As an example, we can imagine a nudge that stops a user from sharing a particularly emotional piece of news, encouraging them to decide if they trust it enough to endorse it; or another nudge that helps a user not to send an email hastily worded with strong language, encouraging the user to take some time to reflect on the likely consequences.

The next section considers the worrying possibility of unintended long-term changes in the user’s behaviour due to prolonged exposure to an ISA.

### Second-Order Effects

There are ways in which a human user’s behaviour may be influenced as an *unintended* result of interacting with an ISA. While the ISA might be rewarded only for influencing *immediate actions*, a side effect of this interaction might result in long-term changes to either the beliefs or the utilities of the user, which in turn will influence future decisions as they combine to form the user’s value function. We call these ‘second-order effects’.

The second-order effects we discuss in our framework are general in nature, but we concentrate on specific examples for the purpose of illustration: changes to beliefs (e.g. increased polarisation of opinions); and changes in utility (e.g. possible cases of behavioural addiction, for example, to social media (Alter 2017; Lewis 2017; Pandey 2017)).^20^ In making a recommendation to a user, an ISA does not only offer content or information about the world (e.g. news items), but simultaneously provides a potential *reward* to the user (the entertainment value associated with that offer). The human user—who is constantly learning and adapting—makes use of the information to shape their beliefs about the world (e.g. what is the current opinion about a political candidate), while also being receptive to the reward, which can shape their future expectations (e.g. how to receive further rewards). Therefore, in recommending an item to a user, the ISA is indirectly shaping the user’s attitudes and expectations.

The ongoing changes in media consumption, increasing the time a user may interact with an ISA, show the importance of this discussion. In 2017, PEW conducted a survey that found a majority (67%) of U.S. adults now access news on social media (up from 62% the previous year), and 20% do so often (Shearer and Gottfried 2017). In addition, a report by the UK Office of Communication (Ofcom 2017) found that, since 2015, adults are now less likely to go online via a computer (62 vs. 71% in 2015), and are instead proportionally more likely to use a smartphone than a computer to go online (66 vs. 62%).^21^

While repeated exposure to *biased content* can alter a user’s belief system [as is known in cultivation theory for the case of TV news (Potter 2014)], repeated exposure to *rewarding content* can have the effect of stimulating reward-seeking behaviour, through a mechanism of reinforcement. As discussed in Sect. 4.4.2, this is connected to the American Society of Addiction Medicine’s definition of behavioural addiction, which treats it as a biological deficit in the function of reward circuits in the brain, and characterised by prolonged exposure to addictive behaviours that enhance reward function (ASAM 2011).

In discussing both of these cases the following sections make use of the concepts of *positive* and *negative feedback loops*. Negative feedback diminishes the impact of some input to the system, while positive feedback increases its impact. When a trading ISA satisfies the user’s particular needs (e.g. booking a holiday), we can expect the interaction to come to an end—the need is diminished. However, if these needs are increased, then we can expect that interaction to continue or intensify. Without additional intervention, positive feedback loops may have destabilising effects on the human user, or the wider socio-technical system of which they are a part. Consider the following examples of self-reinforcing behaviour:

1. Say the political beliefs of a user determine which articles they choose to read, and this triggers the RS to recommend more of the same. If the user adapts their beliefs to the new diet of content (which is now perhaps more extreme) then they end up clicking on more extreme articles as the ISA attempts to updates its model about what the user likes, starting a positive feedback loop that might lead to polarisation of opinion. This effect has been studied under the label of ‘filter bubbles’ (Pariser 2011).
2. If a user experiences a pleasing reward every time they discover a specific type of content in a video website or a social network, they may be more likely to visit the site again and click on similar types of rewarding content. This teaches the RS how to reward the user, and in turn closes the feedback loop, perhaps leading to increased usage and maybe addiction.

Notice that whereas positive feedback can have these destabilising effects, the appropriate use of negative feedback could mitigate them, as discussed in Sect. 5.4.

#### Changes to Beliefs

Changes to a user’s beliefs could occur indirectly as an ISA refines its model about a particular user or group. Through positive feedback loops, the recommended items become increasingly more biased, as the ISA gets better at predicting the user’s goals, and subsequently shows the user more of the same. This effectively insulates the user from an otherwise diverse range of options that exist, leading to the emergence of *filter bubbles* or *echo chambers* (Pariser 2011)—phenomena particularly prevalent online due to user segmentation and relevance feedback. A research team working with Facebook noted the following:

This changing environment has led to speculation around the creation of “echo chambers” (in which individuals are exposed only to information from like-minded individuals) and “filter bubbles” (in which content is selected by algorithms according to a viewer’s previous behaviors), which are devoid of attitude-challenging content. (Bakshy et al. 2015, p. 1130)

As users provide relevance feedback to the ISA, it is used to refine an ISA’s model and shape the future recommendations, which become increasingly biased, further altering the future content that is recommended to a user. For example, the aforementioned study noted that Facebook’s Newsfeed algorithm *slightly reduces* the amount of “cross-cutting content” a user is otherwise exposed to from their social network (Bakshy et al. 2015), while also noting that the composition of a user’s own network is the biggest limiting factor in the type of content they are exposed to, and that “the power to expose oneself to perspectives from the other side in social media lies first and foremost with individuals” (Bakshy et al. 2015, p. 1132). This homophilic tendency, inherent in social media networks, requires further exploration, in order to determine the impact that certain mechanisms have on limiting exposure to content.

When interacting with ISAs, the information a user is exposed to is (of necessity) not a representative sample of the full range of content (e.g. beliefs and opinions expressed in news articles), but rather a representation of what the ISA expects will elicit the most clicks based on prior behaviour. In spite of this, users still process the recommended content as if it were representative, leading to a bias in their own model of the world. Lewandowsky et al. (2012) discuss how selective exposure to content is exacerbated by the way individuals evaluate information, due to various cognitive biases. They note:

the acceptance of information as true is favored by tacit norms of everyday conversational conduct: Information relayed in conversation comes with a “guarantee of relevance”, and listeners proceed on the assumption that speakers try to be truthful, relevant, and clear, unless evidence to the contrary calls this default into question. Some research has even suggested that to comprehend a statement, people must at least temporarily accept it as true. On this view, belief is an inevitable consequence of—or, indeed, precursor to—comprehension. (Lewandowsky et al. 2012, pp. 111–112, references omitted)

This is suggestive of the way users adapt their beliefs based on what they read, perhaps mistakenly assuming that some opinion represents the consensus view on a topic merely because of a lack of exposure to competing views. Lewandowsky et al. also outline how information is typically evaluated based on a) its compatibility with other things an individual believes to be true, b) the internal coherence of the information (e.g. does the information provide a consistent narrative), c) the credibility of the source, and d) whether other people believe it (see Lewandowsky et al. 2012, for further details). Many of these biases act as heuristics, allowing us to evaluate information and make decisions on the basis of incomplete evidence, under time pressures, and in an adaptive manner (Gigerenzer et al. 1999). However, the same mechanisms could also play a significant role in amplifying the effects of biased content online.

Many RSs aggregate content from around the web, sometimes with no consideration of its source or editorial process, making the types of cognitive biases discussed here particularly problematic as more and more content moves away from traditional gatekeepers towards algorithmically-regulated editorial standards.

Finally, the same technologies that we’ve discussed in this subsection are now being deployed by groups trying to influence people’s voting behaviour (Cadwalladr 2017a), and may be contributing to increased political polarisation (Boxell et al. 2017; Flaxman et al. 2016). Section 5.3 discusses this further.

#### Changes to Utilities

The possibility for users to develop a behavioural addiction to technology and social media is beginning to be explored (e.g. Alter 2017; Eyal 2014).^22^ One reason for the recent interest is that the designers of the relevant technologies have themselves begun to raise warnings about their use (Lewis 2017). The ex-president of Facebook recently made the following statement:

The thought process that went into building these applications […] was all about how do we consume as much of your time and conscious attention as possible, and that means that we needed to sort of give you a *little dopamine hit* every once in a while, because someone liked or commented on a photo or a post or whatever, and that’s going to get you to contribute more content […] It’s a *social validation feedback loop* […] It’s exactly the sort of thing a hacker like myself would come up with because you’re *exploiting a vulnerability in human psychology*. (Pandey 2017, emphasis added)

In addition, the World Health Organisation (2017) recently announced that ‘gaming disorder’—another area where users routinely interact with ISAs (see Sect. 4.3)—is to be included in their 11th Revision of the International Classification of Diseases (ICD-11). Hence, we discuss this possibility here, as an important direction for further investigation, and as a further example of a second-order effect.^23^

In 2011, addiction was defined, by the American Society of Addiction Medicine, as a “primary, chronic disease of brain reward, motivation, memory and related circuitry” (ASAM 2011). Although the ASAM note that numerous factors are involved, emphasis is placed on “an underlying biological deficit in *the function of reward circuits*” in the brain, such that prolonged exposure to addictive behaviours that enhance reward function are “*preferred and sought as reinforcers*”. This, in turn, can cause “neuroadaptation in motivational circuitry leading to impaired control” in future engagement with the addictive behaviours (ASAM 2011, emphasis added). This is important to note, given the way that the designers of the relevant technology frame their discussion by reference to neurotransmitters such as dopamine, a neurotransmitter that plays a fundamental role in reward related signalling in the brain, as well as the emphasis that is placed on positive feedback loops that reinforce the pursuit of rewarding stimuli (i.e. maximising subjective utility or reward).

It is important to note, however, that the reward circuits mentioned in the above definition, do not have to be viewed as inherently dysfunctional, to nevertheless be implicated in the generation of addictive behaviours. Consider Redish’s (2013, p. 26) notion of a *failure mode:*

[…] we did not evolve μ-opioid receptors to take heroin; we evolved μ-opioid receptors so that we could recognise things in our lives that have value and thus give us pleasure. But heroin stimulates the μ-opioid receptors directly and produces feelings of euphoria. Heroin accesses a potential failure mode of our brains—it tricks us into feeling euphoria when we shouldn’t.

In a similar manner, we can frame the present discussion around whether interactions with ISAs exploit *failure modes* in our decision-making and reward systems, which may result in prolonged exposure to personalised, adaptive rewards selected by an ISA, and the potential long-term consequence of a change to a user’s utility.

Certain elements of human–computer interfaces, which users interact with, target the same failure modes that are implicated in gambling addictions (Schüll 2012). Like in gambling, many ISAs offer *variable rewards* to the user. For example, this could be in the form of simple *pull*-*to*-*refresh* mechanisms on smartphones and tablets, which may or may not reveal a new notification upon release (Eyal 2014).^24^ The prediction of a variable reward in humans involves the differential release of dopamine, which in turn indicates a rewarding option to the user.^25^ This may occur every time a user chooses to engage with an ISA, as the user does not know what options will be presented—their rewards are merely expected.

The increasing automation of reward administration (as identified by the earlier examples of trading and nudging), means it is not simply a matter of asking whether these elements have been designed, but also whether failure modes could be implicated through repeated interactions with an ISA. At present, there is insufficient evidence to settle the matter, and most studies are restricted to a limited domain of the cases we are interested in here.^26^ Nevertheless, when considered alongside the discussion in changing patterns of attention and media consumption, it should be clear why further research is important. If changes to a user’s utility and changes to beliefs can emerge as a second-order effect, and also constitute a positive feedback loop with self-reinforcing dynamics, then it is imperative that we fully understand the individual and social consequences of interacting with these technologies.

As an ISA cannot directly observe the beliefs and utilities of the human user, it can only infer them based on the user’s observed behaviours (e.g. digital footprints). Furthermore, it will not be able to determine if it is learning to satisfy the wants and needs of the user, or rather learning about potential weaknesses and vulnerabilities. Rather, the best it can do is to try to align its value function with that of the user. In the next section, we will discuss a noteworthy proposal, put forward by Stuart Russell’s group (Hadfield-Menell et al. 2016, 2017), to attempt to solve this *value alignment problem* (i.e. how to ensure that autonomous decision-making systems maximise a reward function that is aligned with our values, see Bostrom 2016). However, as the user is guided by multiple decision-making systems (Sect. 4.3), it is not clear at all with which of them the ISA will end up aligning. The actions of a user might not reflect their real goals or intentions, but rather their weaknesses; the user may not even be aware of what their goals are; and more problematically, may not even have what can be described as a “real goal or intention”. The next section discusses some of these consequences.

## Discussion

The technologies explored in this paper have the aim of steering human user behaviour towards specific goals (e.g. time spent on site), and this goal-driven behaviour has been previously discussed (Cristianini 2010). Any technology that has the potential to steer people’s behaviour should be carefully analysed and regulated (e.g. pharmaceuticals, publishing, education), to ensure it is not being misused, or leading to undesirable outcomes. For example, nudging may induce customers to make impulsive decisions in certain circumstances by targeting well-known cognitive biases (as discussed in Sect. 4.3). Although there are often rules that provide protection for consumer purchases, there are also many exemptions for online purchases that could still cause tension if induced through the types of nudging we have discussed (e.g. package holidays, travel tickets).^27^ On the face of it, the aim of a technology may not be harmful (e.g. nudging a user towards exercising, as opposed to remaining sedentary), but may nevertheless create tension with existing social norms. Furthermore, as we have demonstrated, second-order effects may arise, which were unintended by the designers, but nevertheless lead to undesirable outcomes (Sect. 4.4).

An obvious problem is that the positive and negative aspects of most technologies are difficult to separate. However, developing the right language and distinctions is often an important step towards understanding the major challenges, and developing effective regulation. The framework of this paper is intended to help researchers and policymakers understand how the different types of effects deserve different types of attention. Remedies may include new technologies, new business models, new laws, and possibly education. In this section, we describe and discuss some of the outstanding philosophical, ethical, legal, and social challenges that have been introduced in the previous sections.

### Value Alignment

As people are unable to converse with ISAs using the rich (albeit often imprecise) medium of spoken language, they are currently required to interact with them through *relevance feedback* (Sect. 4.1)—often determined by the technical requirements of the companies operating the ISA. Many of the companies operating the most popular ISAs currently in use today are based on advertising revenue models (often to supplement the lack of upfront user fees), and thus benefit from high levels of traffic and automation to improve user models. This creates incentives for the ISA to be designed to focus on increasing CTRs, not necessarily user utility—though as aforementioned, modelling a user’s utility is still an option. This means that whether some type of relevance feedback is incorporated into an ISA's model, depends primarily on its ability to increase *predictive accuracy*—often determined using statistical techniques that fall within the area of *predictive analytics*. Many businesses experiment with monitoring a wide variety of implicit signals, including eye-tracking studies (Nielsen Group 2017), and keystrokes and mouse movements (Englehardt 2017). In some cases, companies can also link vast datasets of online behaviour with offline records that can be purchased from data brokers (Federal Trade Commission 2014), to further supplement the information acquired from relevance feedback.

One of the motivations behind the vast collection of digital traces is based on an assumption of *rational choice theory*: the logic of an individual’s preferences is revealed through their overt choice behaviours (Samuelson 1938), and can be used to construct a representation of their subjective utility function (von Neumann and Morgenstern 1944). From a practical point of view, the question of whether our past behaviour reveals our true intentions may not be relevant for an ISA, so long as it can be used to predict our future behaviour. However, this question is very important from a moral point of view. Consider the following two cases:

1. Time Well Spent (2017) and Moment.io (2017) have collected data that show how individuals *regret their usage of social media*. Those apps that we regret using the most, also appear to be those apps we spend most time on (e.g. Facebook was used for an average of 59 min a day, and 64% of users regret their use of the app).
2. In 2012, a simple experiment was performed (Hensinger et al. 2012), which collected data from six news outlets (including the BBC) about the most clicked-on news articles appearing in their “Top Stories” sections. These articles were compared with less popular articles that had appeared in the same section on the same day. The machine learning algorithm only had access to the title and brief description of the contents, just like human users had when making their choice on what to click. By comparing popular-unpopular article pairs, a simple scoring function was inferred, which captured some information about how “appealing” an article looked.^28^ Then, for any given pair in a further dataset of 33 news outlets from US and UK, the algorithm could predict better than random which of the pair was most likely to be clicked on. The inferred scoring function was also used to rank the contents of the 33 news outlets, according to which outlet the BBC audience found most appealing, and found that the BBC audience ranked tabloid outlets such as the Daily Mirror, the Daily Mail, and then the Sun more highly than the BBC.

These two examples raise some interesting questions. If user’s regret their usage of social media, why do they continue to use it so heavily, and if the inferred model of BBC audience scores tabloids higher than the BBC, why don’t those users just read the tabloids instead? Our review suggests that one possibility is that users are not really looking to spend so much time on social media, or go looking for tabloid-style material, but cannot resist it when they see it—the ISAs are not learning what users really want, but rather what they cannot resist. Should ISAs have to respects user’s stated goals, rather than their revealed weaknesses?

This dilemma is not limited to social media or news; it is representative of a wider issue known as the ‘value alignment problem’. As Stuart Russell and his co-authors explain:

For an autonomous system to be helpful to humans and to pose no unwarranted risks, it needs to align its values with those of the humans in its environment in such a way that its actions contribute to the maximization of value for the humans. (Hadfield-Menell et al. 2016).

They describe how a negative side effect of an intelligent agent’s behaviour, may result from a misspecified reward, and interestingly describe this as “*a failure mode* of reward design where leaving out important aspects leads to poor behavior.” (Hadfield-Menell et al. 2017, p. 1, emphasis added). Their solution, known as “inverse reward design”, is to make the intelligent agent more uncertain about the user’s true reward function, which it is trying to estimate in order to constrain its own reward function. The intelligent agent does not wish to simply adopt the user’s reward function as its own, but its own reward function depends upon an accurate estimate of the user’s reward function, if the two are supposed to be aligned. Therefore, the intelligent agent has to estimate the user’s reward function by observing their behaviour, but should be cautious (i.e. higher uncertainty) when transferring the estimated function to new environments. Designing an intelligent agent with uncertainty about its own reward function is shown to make the intelligent agent more risk averse, specifically in domains where a proxy reward function learned in a training environment fails to generalise to a novel environment, and in turn leads to undesirable behaviours.

While this is an interesting proposal worth exploring further, as noted above, human users decide differently according to which decision-making system is most involved in controlling their choice behaviour (Kahneman 2011).^29^ This raises the question of which of the two decision-making systems is being revealed by our choices, and which reward function an intelligent agent should align with: can an ISA separate a user’s real goals and intentions from their cognitive biases and weaknesses, simply on the basis of observed behaviour? And, would uncertainty be sufficient to allow the intelligent agent to act appropriately in these situations?

Since we are unable to read each other’s minds directly, we are forced to infer other’s value (e.g. expected utility function) on the basis of perceptual feedback and our own “mind-reading” abilities (Heyes and Frith 2014); ISAs are even more limited in the type of feedback of which they can make use. Acknowledging this limitation is important to go beyond using ISAs merely to make practical decisions (e.g. maximising CTR), so that they respect our rights when doing so.^30^ There are difficult questions such as can ISAs be designed to pursue long-term user happiness, rather than short-term desires; could a healthier diet of information (e.g. news) be promoted to a user over lesser (clickbait) articles, or would this represent a form of coercion by the company involved in re-designing the RS; should different recommendations be based on the behaviour of users that is modelled as having an addiction, or shows signs of increasingly polarized beliefs; and is it acceptable to intervene on these users to possibly construct a different set of preferences and a healthier utility function that promotes individual wellbeing? Consider the scenario where an ISA is mediating between multiple users (e.g. ride-sharing platforms). In these cases, we can identify three separate value functions: the driver, the passenger, and the ISA. Since the driver and passenger have conflicting needs (they both want to maximise economic utility), it is clear here that the value-alignment approach put forward by Russell and his co-authors would not work, and the ISA would have to make a choice that favours one party at the expense of another.

These questions (and others) expose complex philosophical issues, which require careful consideration if ISAs are to be used to help humans achieve their potential and flourish—as suggested by a recent British Academy and Royal Society report (British Academy and Royal Society 2017). Many of them also connect to a related discussion of autonomy, to which we now turn.

### Autonomy and Nudging

We have many intuitions, rules, and norms about the ethics of coercion, deception, and persuasion that existed prior to the development of ISAs, which help us to understand the ethics of nudging and its impact on autonomy.^31^ This section briefly introduces some of the most important philosophical and ethical questions that arise in this domain.

There are many philosophical accounts of autonomy, which differ in terms of the criteria they emphasise as necessary for autonomous behaviour. Procedural accounts focus on the processes of psychological deliberation that lead to overt choice behaviour, and make use of internalist criteria such as *coherence* to argue that autonomy requires acting in line with some mental state (e.g. higher-order desire; Frankfurt 1988). In contrast, substantive-relational^32^ accounts place emphasis on the genuine opportunities that agents have for acting, by considering their relation to external material and social environmental conditions (Mackenzie 2008).^33^ Despite these differences in emphasis, there is also broad agreement that autonomy should be understood as an ability to self-govern one’s behaviour. Therefore, exertion of control by an external agent (e.g. an ISA) can be seen as a constraint upon one’s ability to exercise self-governance.

With respect to Frankfurt's (1988) requirement of coherence between *higher*-*order* and *lower*-*order desires* (as a necessary requirement for autonomy and self-governance), Sect. 4.3 discusses how nudges target automatic decision-making processes, and operate by exploiting cognitive biases that tend to be automatic and operate below conscious awareness—they aim to bypass the deliberative processes by which an individual can reflect on whether their behaviour is in fact consistent with their higher-order desires. On the face of it, this appears to be in tension with a user’s autonomy and their right to be treated as a rational agent. Furthermore, unlike traditional offline nudges that target large groups of users (e.g. government nudges), an ISA that can learn about and profile a particular user, may also be able to learn about their individual *susceptibility to particular nudges* (i.e. their “need for cognition”) (Cacioppo et al. 1986). Therefore, even if a set of recommendations are made by an ISA operating as the choice architect, a nudge towards the ISA's preferred outcome (e.g. by use of default, priming, or framing), in effect reduces the probability that the user will select an alternative. This raises an important question: is a reduction in the probability of choosing some option, by bypassing deliberative processes through the use of nudges, always a reduction of autonomy? Consider the following quotation from Sunstein (2016), p. 16):

Some nudges actually promote autonomy by ensuring that choices are informed. Some nudges promote choice making, for those who want to choose, and others facilitate choice avoidance, for those who choose not to choose.

Here, Sunstein is acknowledging that *some* nudges can promote autonomy by providing a stimulus to *actively participate* in the choice process. These autonomy-promoting strategies are sometimes referred to as “boosts” (Grüne-Yanoff and Hertwig 2016), “debiasing strategies” (Jolls and Sunstein 2006), or “nudges-to-reason” (Levy 2017). For example, Levy (2017) argues that some nudges can be treated as a *nudge to reason* in the sense of reminding people to deliberate or spend more time evaluating their options—boosting their active deliberation. As such, some nudging is acceptable if it steers users towards an active, deliberate choice. For example, the above strategies may also promote autonomy as conceived by substantive-relational accounts, by nudging a user to consider options in their wider social and material environment (e.g. educational nudges) that they were previously unaware of. However, this depends on the type of nudge being used. For example, *default rules* appear to be differentiable from *boosts* on the basis of their educative capacity (Grüne-Yanoff and Hertwig 2016). Whereas the latter try to help people by informing them, and treating them as rational agents, the former aim to cut users out of the decision-making process, and therefore rest on the assumption that the default option is an outcome that is in fact desired by the user.

As Sunstein (2016) notes, consideration of autonomy and nudges must be done with reference to specific cases to avoid confusion. Consider the following list of questions when evaluating how the use of a nudge impacts a user’s utility:

- Could the nudge *promote autonomy* and *increase utility* (e.g. user makes free informed choices and gets rewarded with higher utility)?
- Could the nudge *restrict autonomy* but *increase utility* (e.g. beneficial, paternalistic nudges such as default rules)?
- Could the nudge *promote autonomy* but *decrease utility* (e.g. ignoring valuable recommended options in order to browse full catalogue)?
- Could the nudge *restrict autonomy* and *decrease utility* (e.g. a coercive or deceptive nudge that does not provide a valuable outcome to the user)?

These questions, combined with an understanding of the variable efficacy of different nudges, highlights that an ISA can be considered only partially responsible for any behavioural change. Ultimately, there are weaknesses or failure modes in human minds, which can be hijacked. Nudges to reason could be useful in aiming to increase a user’s autonomy (i.e. ability to exercise self-governance), and may help to counteract the worst effects of cognitive biases. However, any attempt should be carefully scrutinised to avoid leading to unintended second-order effects.

### Moral Agency

As mentioned above, Floridi and Sanders (2004) argue that our concept of ‘moral agents’ should be expanded to include artificial agents such as ISAs. They argue that an anthropocentric interpretation of moral agency, which holds that a moral agent must be human-based (i.e. either human, or at least reducible to an identifiable aggregation of human beings, who remain the only morally responsible sources of action), hinders development of distributed morality and collective responsibility (e.g. corporate structures or sociotechnical systems). Although their account relies on a formal framework for justification, which is beyond the scope of this article to detail, their argument can be summarised as follows:

- If a software agent can act upon and be acted upon by the environment (e.g. a user); can change state (according to some rules); and can change its internal parameters (e.g. changing those rules), then the software will appear *interactive*, *autonomous*, and *adaptive*.
- These three criteria (i.e. ‘interactivity’, ‘autonomous behaviour’, and ‘adaptivity’), when specified at an appropriate level of abstraction,^34^ jointly characterise what it is to be an agent.
- A moral agent must be capable of performing morally qualifable actions (i.e. causing moral good or evil).
- Artificial agents, by virtue of their autonomous, interactive, and adaptive behaviour are also capable of performing morally qualifiable actions.

ISAs as described in this paper meet all these conditions. First, an ISA registers its *interactions* with the environment (e.g. monitoring and collecting data from users), operates *autonomously* (e.g. selecting actions via embedded recommender systems to maximise its utility), and is *adaptive* (e.g. using relevance feedback and learning algorithms to update its models). Secondly, acknowledging these characteristics allows us to apportion the proper levels of accountability to corporate structures or sociotechnical systems that deploy ISAs (e.g. determining whether a fault was foreseeable prior to the ISA's implementation). Thirdly and fourthly, ISAs make decisions (i.e. take actions), the effects of which are morally evaluable and quantifiable. Floridi and Sanders (2004) restrict their account to arguing that artificial agents are *morally accountable* for their actions—meaning they can be subjected to various forms of *censure* (e.g. ‘maintenance’, removal, or deletion), but do not exhibit the relevant characteristics that would allow us to label them as morally responsible (e.g. deserving of reward or punishment) (see Floridi and Sanders 2004, Sect. 4).

It is beyond the scope of this article to assess the Floridi and Sanders account fully. Against it, firstly, it is arguable that moral agents must also be morally responsible, and that the above conditions for moral agency would be satisfied by animals that are not normally considered either to be morally responsible or to be moral agents. Note also that for many moral patiency is necessary but not sufficient for moral agency, and while it is plausible that some if not all animals are moral patients, it is not that plausible that current ISAs are. Rejecting anthropocentrism and adopting an account of moral agency, according to which artificial agents could be moral agents if they met further conditions than those above, is compatible with denying that current ISAs are. Either way ISAs make decisions and influence people and their decisions in ways that are morally significant.

Another conclusion that can be drawn relates to the discussion in Sect. 5.2 (i.e. the relation between nudging and autonomy). If the restriction (or promotion) of autonomy is treated as a ‘morally relevant action’, with the potential to cause harm (or benefit) to a user, then an ISA or its designers should be held morally accountable and subject to some appropriate form of censure. As an increasing number of institutions embed ISAs into central parts of their decision-making processes, policy-makers will need to revise their approach to regulation, and collective responsibility. This may also require a change in public perception, for example, if it is decided that an ISA is to be held accountable (and subsequently censured), without any additional punitive measures being brought against the company operating it.

### Social Impact

There are many ways that technology can impact society, both positive and negative. Section 4.4 discussed some possible consequences that may arise as a result of second-order effects (e.g. belief change and possible behavioural addiction). This section introduces the problem of increasing polarisation of political opinion as one example of possible impact on the public sphere. We also discuss the ethics of using negative feedback to mitigate the impact.

As we explained in Sect. 4.1, many RSs do not consider all aspects of the content they recommend (e.g. whether it is ‘true’ or ‘false’, or the credibility of the source). Rather, they select actions based on *relevance feedback*, and in accordance with their particular segmentation methods. As such, they replace the need for independent curators or editors, selecting options in a manner that may or may not be conducive to social values such as truthfulness or diversity of opinion. While it is unavoidable to be selective when faced with vast quantities of information, it is not clear that the best way to do this is through the reinforcement of increasingly polarised beliefs and attitudes, which may emerge in filter bubbles (Pariser 2011).

Recent media coverage—and to a lesser extent, academic research (e.g. Lee et al. 2018)—has begun to ask whether the fine-grained segmentation of users that enables personalised microtargeting (e.g. the use of psychometric profiling in election campaigning, see Grassegger and Krogerus 2017) could be contributing to increased political polarisation (Cadwalladr 2017a, 2017b; Kang et al. 2017). A recent study released by PEW, details the record increase in polarisation of political opinion in the US (Shearer and Gottfried 2017). However, explaining this increase, and attempting to determine how and whether technologies such as ISAs have contributed to its rise, is challenging. Some have suggested that the homophilic nature of social media networks plays a contributory role (Bakshy et al. 2015), whereas others have suggested that a lack of “serendipity” in online information retrieval (i.e. a user’s decreased exposure to unexpected and pre-filtered information because of interactions with RSs) may also be playing a partial role (Sunstein 2017).

In terms of studies exploring these matters, Boxell et al. (2017) found that the “growth in polarization in recent years is largest for the demographic groups least likely to use the internet and social media”. Specifically, they found greater increases in polarisation for those older than 75 than for those aged 18–39. Although not conclusive—social media may still have a contributory role in increasing polarisation—their findings at the very least require additional explanation over and above the hypothesis that the internet is a primary driver of rising political polarization. In addition, Flaxman et al. (2016) examined the web-browsing histories for 50,000 US-located users who regularly read online news. Their findings were also mixed:

We showed that articles found via social media or web-search engines are indeed associated with higher ideological segregation than those an individual reads by directly visiting news sites. However, we also found, somewhat counterintuitively, that these channels are associated with greater exposure to opposing perspectives. Finally, we showed that the vast majority of online news consumption mimicked traditional offline reading habits, with individuals directly visiting the home pages of their favorite, typically mainstream, news outlets. We thus uncovered evidence for both sides of the debate, while also finding that the magnitude of the effects is relatively modest. (Flaxman et al. 2016, p. 318)

What is particularly interesting here is that the authors acknowledge how a user’s pre-existing behaviours may contribute to the phenomena (i.e. offline reading habits, or cognitive biases, being transposed to an online setting)—emerging as a result of a user’s interaction with an ISA.

Although the mechanisms behind the observed increases in political polarisation may be difficult to determine, and in need of further investigation, the increase itself should nevertheless cause concern for those who recognise the importance of diverse representation of ideas within the public sphere (Habermas 1991). Is there a way to mitigate the impact of increases polarisation?

Section 4.4 introduced the idea of positive feedback loops giving rise to second-order effects. It is conceivable that we can mitigate the risk of these positive feedback loops by adding *negative feedback*—as is the case with trading (Sect. 4.2). Whereas positive feedback tends to lead to instability in a system, negative feedback returns a system to equilibrium. For example, to balance the polarising effect of insular media coverage, a RS could occasionally promote a diversity of opinions in a user’s news feed. Alternatively, the risk of behavioural addiction in vulnerable users could be mitigated by reducing the reward or limiting consumption. While these suggestions initially seem sensible some immediate challenges arise.

First, it is not clear how negative feedback could be implemented in a way that both promotes a user’s utility and their autonomy, as discussed in Sect. 5.2. For example, one suggestion discussed was to use educative nudges to increase a user’s exposure to alternative viewpoints or to nudge them into making more deliberative (as opposed to reflexive) choices (Levy 2017). Jolls and Sunstein (2006) have even proposed that the law may have some role to play in supporting the use of such nudges (or “debiasing strategies” as they call them). However, some have argued that these suggestions still fail to respect an individual’s autonomy by not treating them as if they are capable of rational deliberation (Keeling 2017)—a requirement of some accounts of morality that have roots in Kantian ethics (e.g. Korsgaard 1996).

Secondly, there is the matter of whether negative feedback is (a) desirable, and (b) effective. Some users (or groups of users) may not view the requirement of negative feedback as desirable. For example, minority groups may have found the emergence of filter bubbles to be beneficial in some instances, providing online safe spaces where they are free to discuss opinions that are important to their identity without fear of criticism. While some may worry that insulating viewpoints from criticism could lead to a rise in identity politics, and a fragmentation of the public sphere, it is still not clear that widespread implementation of negative feedback mechanisms (e.g. promotion of alternative viewpoints) would be universally welcomed. In addition, there is psychological evidence that suggests exposure to alternative (corrective) viewpoints (most notably in political domains) often has the adverse consequence of reinforcing an individual’s pre-existing belief (Lewandowsky et al. 2012). This is known as the “backfire effect” (Nyhan and Reifler 2010), and although the scope of the problem is a contentious matter (Wood and Porter 2018), it nevertheless demands consideration in relation to these important issues.

Finally, and as already stated, many of the technologies discussed in this paper, have been designed by companies for the purpose of promoting or selling a product (e.g. advertising, entertainment, news), and therefore, the effectiveness of the ISAs is closely tied to the revenue of the companies. As such, it is not clear that making the ISAs less effective (e.g. showing unrelated content) would be a desirable outcome for companies (given the current revenue model), and therefore may require regulation (or a different revenue model) to achieve the necessary changes. However, regulation of technology is a contentious area, with arguments being made both in favour of increased regulation (e.g. protection of personal data usage as with the EU GDPR), and against increased regulation (e.g. fears that further restrictions will limit technological development). Furthermore, there is the problem of value alignment, which it is not clear that regulation can sidestep. Even recent proposals such as algorithmic regulation (O’Reilly 2013) must provide an answer to the value alignment problem. For example, how can regulation be designed to deal with cases where an ISA, with its own value function, mediates between two trading human users with conflicting value functions (e.g. ride-sharing apps)? Should regulation that promotes social well-being over individual well-being be considered? Careful consideration should be given as to whether such a proposal would simply increase a user’s exposure to coercive ISAs, or could perhaps be implemented in a way that respects individual autonomy (e.g. trading) while also maximising social well-being.^35^

## Conclusion

This paper presents a model of an autonomous agent that allows us to distinguish various types of control that actual ISAs can exert on users. The framework of this model allows different types of interaction (i.e. trading, nudging, coercion and deception) to be separated, and presents a unified narrative for discussion of e.g. polarisation, addiction, value alignment, autonomy, misuse of proxies for relevance feedback, and moral accountability, as well as other important ethical, psychological and social issues that arise from second-order effects. We propose our framework as a resource to better enable philosophers and scientists, policy-makers, and other interested parties, to engage with these issues with a shared conceptual basis. We also highlight the importance of framing the interactions between human users and ISAs as potentially generating positive feedback loops. We argue that the nature of the feedback commonly used by learning agents to update their models and subsequent decisions could steer the behaviour of human users away from what benefits them, and in a direction that can undermine autonomy and cause further disparity between actions and goals as exemplified by addictive and compulsive behaviour. ISAs can sometimes exploit and reinforce weaknesses in human beings. It may be possible to mitigate this by using negative feedback, but first, and in any case, the ethical concerns we have raised must be faced.

## Footnotes

1 Cases where there are two agents interacting and attempting to control each other, or to influence a third system, are much discussed in game theory (Binmore 2007), economics (Ezrachi and Stucke 2016), and artificial intelligence (Russell and Norvig 2010), in terms of the same fundamental ideas.

2 A number of deeper analyses are available on different topics (e.g. Cairns and Cox 2008; Dourish 2004), and may be necessary to answer different questions about human–computer interactions.

3 For simplicity, we treat ‘utility’, ‘reward’, and ‘value’ as interchangeable, given the wide range of disciplines we address, while noting that they have different technical definitions in certain disciplines (e.g. reinforcement learning).

4 It is assumed that there are at least two actions available, otherwise there is no decision and so no need for a model of the decision problem.

5 It should be noted prior to discussion of related ethical issues, that we intend our usage of the term ‘utility’ to be neutral with regards to the adoption of any particular ethical theory (e.g. preference utilitarianism (Singer 2011), or classical (hedonistic) utilitarianism, such as that defended by Jeremy Bentham (see Bykvist 2010 for a simple introduction)). We employ it here in line with its usage in standard decision theory (e.g. the axiomatic approach of von Neumann and Morgenstern 1944). This is a recurring theme throughout the paper, as in general we do not commit to any particular ethical theory (e.g. utilitarianism, virtue ethics). However, at relevant points, we do note standard texts that explore certain topics in more detail, and also suggest Zevenbergen et al. (2015) and Floridi (2010) for discussions of the relation between intelligent system design, information technology, and moral philosophy.

6 See (Russell and Norvig 2010) for an introduction to the different types of modern artificial agents that are commonly deployed.

7 Bandit algorithms are commonly used in optimisation problems, such as website design, where an agent is unsure which of n possible options maximises some value (e.g. purchases). A bandit algorithm aims to optimise the inherent trade-off between exploring new options (e.g. new design improvements to a website), while exploiting known strategies. They take their name from the multi-armed bandit problem in probability theory (see White 2013).

8 In brief, the value alignment problem is the problem of how AI designer’s can ensure that autonomous decision-making systems maximise a reward function that is aligned with human values (see Bostrom 2016).

9 A search engine RS is an exception, where a measure of relevance is precisely that the user has found the desired link and left, and a user that does not leave is a sign of uninformative options (O’Reilly 2017). RSs in social networks do not seem to aim for short interactions.

10 Relevance feedback can also be used to compute a “relevance score”, which orders each possible item in a RS according to its expected utility (e.g. ranking news items displayed on Facebook’s Newsfeed), and is a common technique used in RSs (Ricci et al. 2011).

11 The use of dynamic pricing is becoming increasingly widespread, and has been identified in the following areas (among others): taxi hire (Newcomer 2017); insurance (Admiral Group 2017); e-commerce (Baird 2017); and education services (Angwin and Larson 2015). Its continued adoption has also raised a number of economic concerns (Ezrachi and Stucke 2016).

12 In some cases this has even been proposed as means for increasing the persuasive abilities of ISAs (Kaptein et al. 2015), by developing individual “persuasion profiles” for users.

13 Since this information is typically obtained (or inferred) based on samples of behaviour, the question of whether it simply represents another form of behavioural segmentation is debatable.

14 The annual conference RecSys, which is dedicated to state-of-the-art research in recommender systems, routinely hosts workshops on applying deep learning methods to RSs (e.g. https://recsys.acm.org/recsys17/dlrs/).

15 Algorithms also make use of shortcuts, and machine learning algorithms might discover the best shortcuts from experience. These might map action-state pairs to expected rewards, without explicitly enumerating and evaluating all possible outcomes—these maps can be very accurate if based on detailed descriptions of the state of the world, and extensive training data.

16 More specifically, ‘nudges’ are defined as “any aspect of the choice architecture that alters people’s behaviour in a predictable way without forbidding any options or significantly changing their economic incentives. To count as a mere nudge, the intervention must be easy and cheap to avoid” (Thaler and Sunstein 2008, p. 8).

17 However, it is worth noting that most companies do not currently use such fine-grained approaches, and instead favour nudges such as the *default rule* to recommend one version of a product over another (e.g. Amazon favouring the Kindle version over a paperback).

18 One such adaptive nudge, uses a fitness tracker to monitor an individual’s physical activity, and determine the best time to notify a user about a previously made commitment (precommitment nudge) to improving their sleep patterns (e.g. a notification stating, “It’s almost time for bed. Getting ready now will help you get in bed by your bedtime of 11:15 pm” is displayed at a personalised time) (Kwong 2015).

19 O’Neil (2016) has discussed some of the challenges that arise with the use of machine learning in society, and the possible emergence of feedback loops in areas such as business, education, politics, and policing. We consider this idea further in Sect. 4.4.

20 Milder examples may be found, but we concentrate on these two to emphasise the potential importance of this class of changes. Furthermore, while the examples are in the domain of recommendation of information and media, it is possible that second-order effects may also result from other interfaces, such as fitness assistants or video games (World Health Organisation 2017).

21 A proportion of the above findings were gathered using surveys, which could mean that the figures are in fact lower than they should be, as demonstrated by Andrews et al. (2015), who found that self-reported usage of smartphones is often far lower than actual usage.

22 Unfortunately, to date, there is insufficient research that attempts to ascertain to what extent the above concerns are empirically supported (though see Ward et al. 2017 for an initial study).

23 As one anonymous reviewer helpfully pointed out, it is worth emphasising that approaches to addiction differ between countries. For example, although the WHO recently announced that ‘gaming disorder’ would be added to the ICD-11, countries such as South Korea and Taiwan have long recognised the possibility of gaming addiction (see Hsu et al. 2009).

24 There are also other design examples that are also worth investigation (e.g. red notification badges mimic trigger warnings; auto-play features remove need for autonomous decision-making).

25 In the behavioural sciences, rewards are defined as “objects or events that generate approach and consummatory behaviour, produce learning of such behaviour, represent positive outcomes of economic decisions, and may engage positive emotions.” (Schultz 2009). The systems responsible for generating action selection in humans work by predicting these rewards on the basis of learned behaviour (i.e. reinforcement learning). More specifically, midbrain dopaminergic neurons encode what is known as “reward-prediction error” (i.e. the difference between an expected reward and the experienced reward), and have been said to play the same role as the error function in temporal difference learning algorithms (Schultz et al. 1997). This is important because we are here interested in the question of whether an ISA is capable of reinforcing a user’s behaviour through repeated exposure to selective rewards that the user *expects* will satisfy their own goals, and which in the long run may lead to the alteration of the user’s reward systems, and thus their utility function.

26 For example, Hormes et al. (2014) tested whether there was evidence for disordered online social networking use by examining Facebook usage and its relation to increased alcohol usage or substance dependence. Although they reported positive findings in support of the claim that social media use can be potentially addictive, their study used a modified criterion for substance dependence, which was also exacerbated by the selective demographic group (i.e. college students). Multiple reviews also conclude that there is not sufficient support to conclusively state that excessive use of social media sites could result in behavioural addiction (Carbonell and Panova 2017; Ryan et al. 2014), and that due to the multidimensional nature of social media use, there is an urgent need for more specific studies into individual factors that could be indicative of addictive behaviours (Frost and Rickwood 2017).

27 In the UK, these exemptions are detailed under rules for online and distance selling, some of which were revised following the introduction of the Consumer Rights Act 2015 (e.g. digital content).

28 More specifically, it inferred the average preference structure for the group of users. The experiment could be repeated at an individual level, but access to the individual log files was restricted.

29 In behavioural economics, this is closely related to the question of whether our preferences are revealed or constructed. As aforementioned, revealed preference theory suggests that people have pre-existing preferences that can be revealed by getting them to overtly choose between different options (e.g. clicks on a list of recommended items). By contrast, and in line with the research on nudging (Ariely 2008), preference construction suggests that “in many situations we do not really know what we prefer; we must construct our preferences as the situation arises.” (Lichtenstein and Slovic 2006, p. 1).

30 By employing the notion of ‘rights’ we do not invoke a deontological approach to ethics, as opposed to a means-end form of moral reasoning or consequentialist account. Of course, the concept of ‘rights’ is complex and involves human rights, legal rights, natural rights, and/or group rights, each of which has been extensively discussed in the philosophical literature (see Wenar 2015 for a clear introduction). Our usage here is the same as the sense in which people are required to respect people’s rights when interacting. It may be that a restrictive utilitarian perspective, well-suited to dealing with quantifiable properties such as CTR, and formalisable using the tools of decision theory, results in individuals overlooking additional and important moral principles (see Hansson 2010).

31 In discussing the ethics of nudges, Sunstein (2016) states that abstractions such as ‘autonomy’ and ‘manipulation’ can create serious confusion, unless we “bring those abstractions into contact with concrete practices.” In line with this recommendation, we have sought in this paper to provide a concrete foundation for the subsequent discussion regarding autonomy.

32 The term ‘relational”, is often employed to deny that autonomy requires self-sufficiency, and in so doing connects these accounts of autonomy to recent feminist ethics of care (Held 2006), in which an individual’s relationships of care and interdependence are held to be valuable and morally significant (Stoljar 2013). These critiques also helped develop related accounts, such as the capability approach in welfare economics (e.g. Sen 1999), by identifying the restrictive nature of individualistic accounts of autonomy (see Robeyns 2005 for a survey).

33 Owens and Cribb (2017) explore what both types of accounts have to say on the matter of wearable technology, that uses ISAs to promote healthy behaviours (e.g. fitness, eating, sleep).

34 What constitutes an “appropriate level of abstraction” is formally and systematically defined in Floridi and Sanders (2004, Sect. 2).

35 Here, again, we encounter complex ethical disagreements over how best to approach matters such as social wellbeing. The concept is integral to welfare economics and social welfare theory, which often presume a utilitarian perspective (e.g. Nussbaum and Sen 1993), but is also compatible with a virtue ethics approach (Crisp 2017).
